# Accuracy of emergency physicians’ self-estimates of CT scan utilization and its potential effect on an audit and feedback intervention: a randomized trial

**DOI:** 10.1186/s43058-021-00182-1

**Published:** 2021-07-27

**Authors:** Celine Larkin, Alexandra M. Sanseverino, James Joseph, Lauren Eisenhauer, Martin A. Reznek

**Affiliations:** University of Massachusetts Medical School, Worcester, USA

**Keywords:** Audit and feedback, Physician ordering, Emergency medicine

## Abstract

**Background:**

Audit and feedback (A&F) has been used as a strategy to modify clinician behavior with moderate success. Although A&F is theorized to work by improving the accuracy of clinicians’ estimates of their own behavior, few interventions have included assessment of clinicians’ estimates at baseline to examine whether they account for intervention success or failure. We tested an A&F intervention to reduce computed tomography (CT) ordering by emergency physicians, while also examining the physicians’ baseline estimates of their own behavior compared to peers.

**Methods:**

Our study was a prospective, multi-site, 20-month, randomized trial to examine the effect of an A&F intervention on CT ordering rates, overall and by test subtype. From the electronic health record, we obtained 12 months of baseline CT ordering per 100 patients treated for every physician from four emergency departments. Those who were randomized to receive A&F were shown a de-identified graph of the group’s baseline CT utilization, asked to estimate wherein the distribution of their own CT order practices fell, and then shown their actual performance. All participants also received a brief educational intervention. CT ordering rates were collected for all physicians for 6 months after the intervention. Pre-post ordering rates were compared using independent and repeated measures *t* tests.

**Results:**

Fifty-one of 52 eligible physicians participated. The mean CT ordering rate increased significantly in both experimental conditions after the intervention (intervention pre = 35.7, post = 40.3, *t* = 4.13, *p* < 0.001; control pre = 33.9, post = 38.9, *t* = 3.94, *p* = 0.001), with no significant between-group difference observed at follow-up (*t* = 0.43, *p* = 0.67). Within the intervention group, physicians had poor accuracy in estimating their own ordering behavior at baseline: most overestimated and all guessed that they were in the upper half of the distribution of their peers. CT ordering increased regardless of self-estimate accuracy.

**Conclusions:**

Our A&F intervention failed to reduce physician CT ordering: our feedback to the physicians showed most of them that they had overestimated their CT ordering behavior, and they were therefore unlikely to reduce it as a result. After “audit,” it may be prudent to assess baseline clinician awareness of behavior before moving toward a feedback intervention.

Contributions to the literature

- Audit and feedback (A&F) is a strategy used to help clinicians understand and modify their behavior.
- Few previous studies have examined whether the clinicians’ baseline estimates of their clinical behavior relate to the effectiveness of audit and feedback.
- We show that emergency physicians overestimated their CT ordering behavior and that an A&F intervention for that target behavior was ineffective.

## Introduction

Computed tomography (CT) is an important diagnostic tool in the armamentarium of emergency medicine (EM) physicians as they care for patients. First implemented in a limited number of hospitals in the 1970s, CT utilization initially grew slowly [1]. However, by the 1990s, CTs were widespread and utilization expanded quickly. In fact, emergency department (ED) CT utilization increased by 330% from 1996 to 2007 [2] and has continued to grow since [3].

Multiple studies have demonstrated high variability in CT utilization both within and across institutions [4–7]. Variability in the utilization of any testing modality has significant negative efficiency and cost implications [8], but CT use also has patient safety implications, given that ionizing radiation places patients at increased risk of developing cancer [9]. These safety concerns have prompted multiple national bodies to implement evidence-based campaigns to reduce unnecessary CT utilization [10].

In recent years, it has come to light that ED physicians may lack self-awareness of their own CT utilization behaviors [11], which could be contributing to the observed variability in ordering practices. Given the poor self-awareness reported to occur in ED-based CT ordering, audit and feedback (A&F) may be an effective implementation strategy to improve physicians’ CT ordering behavior. A&F, the technique of providing a summary of performance over a specified period of time to enable individuals to self-assess and adjust their performance [12] to better align with peer norms or benchmarks, has been shown in meta-analyses to have a modest to moderate effect on healthcare provider behavior [13]. A&F in healthcare settings is often theorized to work by improving clinicians’ accuracy in estimating their own performance [14, 15], but few A&F studies actually have measured that accuracy. In 2018, Michael et al. [16] found that EM provider self-awareness of practice behaviors was a significant factor in behavioral change related to opioid prescribing. Their randomized, controlled trial showed that an A&F strategy, when added to augment local and state-wide prescription opioid educational efforts, resulted in a greater reduction of opioid prescribing in those EM providers who underestimated their pre-intervention prescribing behaviors.

Perceiving potential similarities with EM opioid prescribing and EM CT utilization (over-prescribing and over-ordering, respectively) as well as potential similarities in limited self-awareness in both EM opioid prescribing and CT utilization [11, 16], we postulated that a similar implementation strategy as reported by Michael et al. for opioid prescribing may similarly result in CT utilization reduction. Therefore, in the same study setting and with a similar methodology as the aforementioned opioid investigation, we undertook an A&F investigation related to ED CT utilization. As an exploratory aim, we measured the clinicians’ estimates of their own performance prior to the intervention, considering the importance ascribed to self-awareness as a mechanism of A&F effectiveness.

## Methods

### Trial design

Our study was a prospective, multi-site, 20-month, randomized trial to examine the effect of an A&F intervention on CT ordering rates. Study participants were randomized to one of two arms: the A&F intervention arm or the control arm. Subjects in the intervention arm participated in an individual, in-person review session with a research assistant to (1) estimate their own CT ordering rate compared to other physicians in the study and (2) view their actual CT ordering rate compared to other physicians (described in more detail below). CT ordering rate was displayed as the number of CTs ordered per 100 patients seen. Subjects in the control condition did not receive this intervention. All participants received a one-off brief didactic on the risks of CT overuse. By necessity, participants and the research assistant were not blinded to condition assignment, but those extracting and compiling outcome data were blinded to condition assignment. The study was approved by the University of Massachusetts Medical School Institutional Review Board (IRB).

### Study setting and participants

Eligible study participants included board-certified or board-eligible EM physicians who practiced in the ED of one or more of four separate hospitals that were part of a single, larger health system, with approximately one-third of the physicians practicing at a single study site and two-thirds practicing at multiple. The four study sites included a 364-bed urban tertiary care, academic center (92,000 annual ED visits); a 294-bed urban acute care hospital/non-primary teaching site (42,000 ED visits); a 69-bed suburban community hospital (26,000 ED visits); and a 41-bed rural community hospital (14,000 ED visits).

All ED patients at the four sites were evaluated and treated by an attending physician. At the academic center, approximately 80% of ED patients were also evaluated and treated by residents—physicians receiving specialized, supervised training following medical school, generally in pursuit of board certification. At a secondary teaching site, residents participated in the care of fewer than 5% of patients. Residents were not included in this study because their practice of ordering CTs was directed by a supervising attending physician present at any given time, and the residents’ practice was therefore reflective of the attendings’ practice preferences rather than their own. Advanced practice providers (physician assistants and nurse practitioners) were also employed within the department; however, they practiced primarily in an observation unit and a low-acuity patient care area. In this capacity, they rarely ordered CT scans, so we did not include APPs in this investigation. At the primary teaching site, acute trauma patients were comanaged by the ED attending physician and a trauma team, so CTs ordered for patients being comanaged were excluded as they did not necessarily reflect the ordering behavior of the ED attending, and rather than that of the trauma team.

Eligible physicians were invited to participate in the study if they had been working within the department throughout the 12-month baseline observation. Subject participation was voluntary. Participants were excluded post hoc if they did not remain for the 6-month follow-up observation period.

### Intervention

We hypothesized that A&F intervention would reduce EM physician CT ordering behavior by improving their understanding of their own ordering behavior and that of colleagues. To create A&F intervention instruments, we extracted CT ordering data from the institutional electronic health record (EHR) (Epic, Epic Systems Corporation, Verona, WI, USA) for each eligible physician over a 12-month period (June 1, 2018, to May 31, 2019) prior to the intervention. We calculated for each provider the total number of CT scans ordered per hundred patients cared for by that provider as well as for subtypes of CTs including abdominal/pelvic CT, head CT, cervical spine CT, and chest CT for pulmonary embolism. We then constructed graphical depictions (Appendix) of the distribution of CT ordering per 100 patients, including data from all providers eligible for randomization, based upon the ordering data for the 12 months prior to randomization. (The completeness and reliability of this data were high given that the treating physician either personally placed the CT order within the EHR or, in the case that a resident physician ordered the CT, it was automatically attributed within the EHR to the attending physician who was directly supervising the resident at the time.)

To randomize subjects, we performed stratified permuted block randomization using a computerized engine to allocate providers to control or intervention arms in a 1:1 ratio. We stratified the randomization by quartiles of baseline ordering, measured by the number CT scans ordered by each provider in the prior year. A trained research assistant approached each provider randomized to the intervention arm on the ED floor before or after a patient care shift or in an administrative area and performed the brief A&F intervention once by privately showing the provider the anonymized graphical distributions, which depicted the baseline distribution of CT ordering by all providers. Scripting explained, “Each of these bars represents one provider in the group, including you. Which do you think is you?” The assistant then asked the participants to self-identify their estimated individual position, which the research assistant recorded as a decile with reference to the group. Immediately after the above inquiry, the staff provided the participant with feedback regarding their true position relative to group norms, including absolute utilization data and a visual display of where they fell within the peer distribution. Controls were not surveyed as to their self-perception and did not receive individual or group data. The groups were treated similarly in all other respects, including a concurrent, department-wide educational effort aimed at reducing CT utilization by discussing CT-associated risk of cancer and nationally accepted standards for optimal CT ordering. Participants in both arms were expected to participate in a 30-min educational session that was primarily didactic, with opportunities for learner interaction. For physicians who could not arrange to participate in a face-to-face didactic session, the associated slide presentation was provided to them, and they were expected to review the slides. We held the education session during a regularly scheduled staff meeting because physicians generally derived value from the staff meetings and felt a sense of accountability for information shared during staff meetings as part of the local professional culture. For similar reasons, physicians generally studied the minutes and any associated attachments from the staff meetings, so we perceived a higher likelihood that those that were not able to attend the educational session would study the slides provided via the meeting minutes. Notably, within the CT utilization education session and the A&F intervention, no absolute utilization goals were suggested because there are no evidence-based specific standards. Rather, subjects were presented evidence that EM physicians are known to utilize CTs outside of evidence-based criteria for certain clinical indications resulting in over-ordering of CTs, coupled with available evidence related to risks associated with ionizing radiation for CT scans.

We employed a 2-month (June 1, 2019, to August 5, 2019) transition period between the pre- and post-intervention observation blocks to allow for subject randomization, for all participants to be able to complete the CT education program and for time to complete the A&F intervention with all subjects in the intervention arm. For the subsequent 6 months (August 6, 2019, to February 5, 2019), we passively observed prescribing patterns electronically.

### Outcomes

The primary outcome was a change in physician CT ordering rates per 100 patients seen following the A&F intervention, including overall CT ordering rate and ordering rate for the following subsets of CTs: CT abdominal/pelvic, CT head CT, CT cervical spine, and CT chest with contrast for pulmonary embolism CT. As an exploratory aim, we also measured the intervention arm participants’ actual versus self-perceived CT ordering behavior prior to the intervention, based on where on the curve the participant placed their estimated individual CT ordering position.

### Statistical analyses

While we hypothesized that A&F would reduce CT ordering in the intervention group, this type of intervention had not been reported for CT ordering before, so we conducted all tests as two-sided. We examined pre-post changes in overall and subtype CT ordering within each group using repeated measures *t* test and employed linear regression to examine the effect on post-intervention ordering controlling for pre-intervention ordering. We considered subjects’ estimates of their ordering behavior to be inaccurate (an overestimate or underestimate) if it was not in the correct decile. We used one-way analyses of variance to compare the rates of ordering across intervention arm clinicians who under-, over-, and accurately estimated their own CT ordering rate. The significance level was set as *p* < 0.05, and all analyses were conducted with SPSS version 27 (IBM, Armonk NY, USA).

## Results

A total of 52 physicians were eligible and invited to participate, of whom 51 volunteered. Twenty-five physicians were randomized to the active A&F condition, and 26 were randomized to the control condition. The intervention and control arms differed slightly by gender, with a lower proportion of men in the intervention (52%) versus the control (69%) group. However, the baseline mean CT ordering rate seen was comparable in each arm (intervention, 35.7/100 patients; control, 33.9/100 patients). Fifteen (60%) A&F arm subjects and 11 (42%) control arm subjects participated in the in-person education session. All study participants received the slides associated with the CT education session.

### Accuracy of self-estimation

**Fig. 1:**
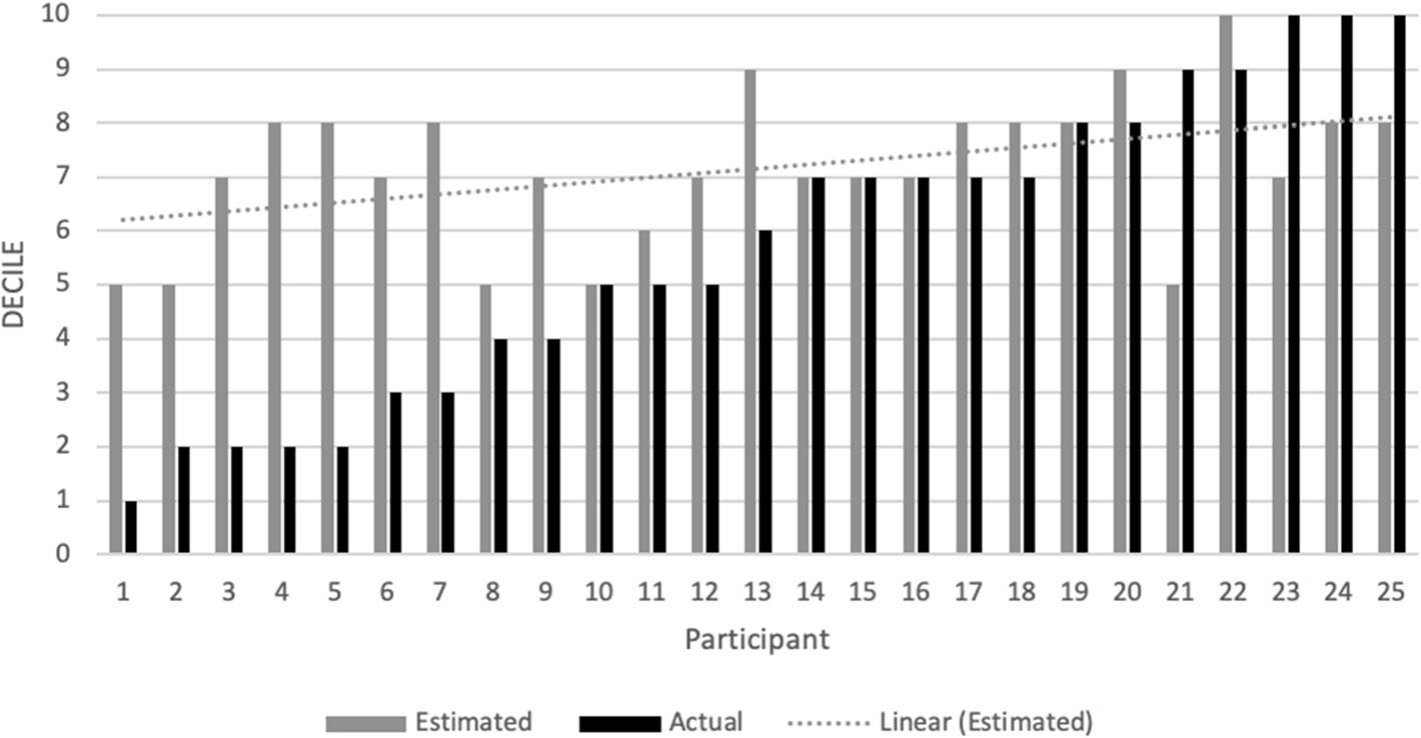
Self-estimated and actual decile of each physician’s CT ordering before the A&F intervention

None of the participants in the intervention arm estimated their CT ordering rate to be within the lower five deciles (bottom half) of CT ordering in their peer group. Sixteen physicians (64%) overestimated their decile of CT ordering by at least one decile, four physicians underestimated (16%), and five guessed accurately (20%). Those who overestimated did so by a median of 3 deciles (IQR, 1–4.75) (Fig. 1).

### Effect of intervention on CT ordering

**Fig. 2:**
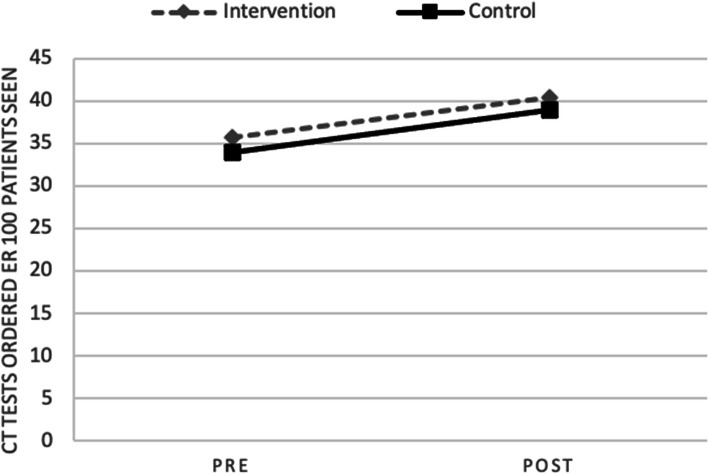
Rates of CT ordering per 100 patients increased significantly in both the intervention group and the control group between baseline and follow-up

Rates of CT ordering per 100 patients increased significantly in both the intervention group and the control group between baseline and follow-up (Table 1 and Fig. 2), with no significant between-group difference observed between the two arms in the follow-up period (*t*(45) = 0.43, *p* = 0.67). (With alpha set at 0.05, our sample size of 51 conferred 0.8 power to detect a moderate to large effect size (Cohen’s *d* = 0.7).) Head CT ordering increased significantly in the intervention group, and most other CT ordering trended upwards in both groups over time for both intervention and control arms. Controlling for pre-intervention CT ordering, there was no effect of the experimental group and post-intervention CT ordering (beta *p* = 0.02, *p* = 0.80).

**Table 1:** CT scan ordering rates by scan subtype and experimental group in the pre- and post-intervention periods

	Intervention condition				Control condition			
	Pre	Post	*t*	*p*	Pre	Post	*t*	*p*
*N ordered per 100 patients seen*	*m (SD)*	*m (SD)*			*m (SD)*	*m (SD)*		
All CTs	35.7 (10.0)	40.3 (11.8)	4.13	**< 0.001**	33.9 (9.2)	38.9 (11.9)	3.94	**0.001**
Abdominal/pelvic CT	10.6 (2.9)	10.8 (3.3)	0.56	0.58	9.3 (2.7)	9.5 (3.0)	0.82	0.42
Head CT	9.4 (3.2)	10.9 (3.6)	2.22	**0.04**	9.7 (2.6)	10.5 (3.0)	1.78	0.09
Spine CT	3.5 (1.9)	3.5 (1.5)	-0.02	0.99	3.4 (1.4)	3.5 (1.5)	0.67	0.51
Pulmonary embolism CT	2.5 (1.1)	2.6 (1.3)	0.31	0.76	2.3 (1.0)	2.5 (1.1)	1.52	0.14

### Association between self-estimate and change in ordering

There was only a moderate correlation between estimated and actual decile of CT ordering behavior compared to peers (*r* = 0.31, *p* = 0.13). Those who underestimated their rate of ordering had placed significantly more CT orders per 100 patients in the pre-intervention period (m = 52.7, SD = 5.5) than those who estimated accurately (m = 37.1, SD = 2.0) or those who overestimated (m = 30.9, SD = 7.0; *F* = 19.96, *p* < 0.001). CT ordering increased in all self-estimate accuracy groups in the post-intervention period: the increase was slightly more marked in those who had overestimated or were accurate at baseline but the magnitude of the increase was not statistically different across the groups (*F* = 0.52, *p* = 0.60).

## Discussion

The results of our investigation revealed that an A&F intervention did not reduce CT ordering rates for ED physicians. Ours is the first study to test an A&F intervention on ED CT utilization using an RCT methodology, so there are no directly comparable investigative reports for us to consider when evaluating our results. However, considering our results in the context of a previous ED provider A&F investigation, which had a similar intervention methodology and similar study setting and population, appears to provide some important insights.

Our investigation uncovered relatively poor physician self-awareness of CT ordering behavior, with only 20% of physicians accurately assessing their practice and most physicians overestimating their own CT ordering rate. Based on a previous study of CT ordering self-estimates, we had expected physicians to underestimate their CT ordering frequency [11]. We had expected our A&F intervention to work by demonstrating that underestimation to physicians, thereby enhancing their self-awareness of actual ordering behavior, and prompting them to reduce ordering. Given that their estimates were instead characterized by significant overestimation, it is possible that this overestimation is a contributing factor to the lack of effect of our A&F intervention.

In the Michael et al. investigation of ED opioid prescribing, investigators found that A&F altered ordering behavior for providers who underestimated their prescribing behavior compared to group norms, with no statistical difference for those that overestimated their prescribing compared to the control group [16]. Considering the results of our investigation in conjunction with the prior ED opioid A&F study, it appears possible that the pre-intervention self-perception of providers undermines the effectiveness of an A&F intervention in reducing CT scan ordering. This notion that A&F may not be effective, or even deleterious, in altering behavior in a desired direction if the subject presumes at baseline that their behavior is “worse” than peers is consistent with the feedback intervention theory (FIT) proposed by Kluger and DeNisi, in which they emphasize the importance of feedback drawing attention either toward or away from a task [17]. It is possible that our feedback intervention drew subjects’ attention away from the task of modulating CT ordering when they learned that they were actually ordering fewer CT compared to group norms than they originally believed. This issue of attention may be especially salient in emergency medicine, with its inner context characterized by many competing and urgent demands, high physician autonomy, and an emphasis on efficiency and flow [18, 19]. This implies that those seeking to use A&F to change behavior should first seek to understand subjects’ perceptions of their behavior and perhaps consider a strategy of “audit and reflect” before giving feedback to ensure that the presented data will have the desired effect on the intended group and/or individuals. Certainly, additional research is required into the accuracy of self-estimates of a range of clinical behaviors, whether asking clinicians to estimate their behavior is an intervention in itself, how the accuracy of self-estimates may affect the effectiveness of data-driven implementation strategies like A&F, and whether these clinical findings are generalizable to non-clinical settings.

It is important to note that in the education we provided to all physicians, we did not set specific goals for CT ordering because there were no evidence-based benchmarks for utilization rates. We therefore took the approach of presenting an evidence-based rationale for the need to reduce CT ordering by following established guidelines for appropriate CT ordering in order to improve patient safety. Those guidelines were limited to specific clinical scenarios that can be difficult to audit for compliance [20–22], thereby limiting their utility in objective measurements related to CT ordering behavior. In the referenced opioid A&F investigation [16], the educational intervention did include specific opioid prescribing practice goals mandated by the state that were easily auditable. It is possible that the lower specificity in behavioral expectations and potentially perceived lower stakes in our investigation compared to those in the opioid investigation contributed to our observed results.

Another potential contributor to the observed differences between our investigation and opioid A&F study was the potential that providers perceived more serious consequences to underordering CT scans as opposed to underordering opioids. Underordering CT scans had the risk of leading to missed diagnoses potentially harming patients or resulting in harm to the physician themselves via malpractice claims, as opposed to underordering opioids which risked inflicting undue discomfort to the patients and lower patient satisfaction ratings of the physician. The perceived risks associated with underordering CTs may have been a greater deterrent than that for opioids, thereby potentially influencing our observed results. A final consideration with respect to our comparing the outcomes of our investigation with that of Michael et al. was that the investigations were performed with a 15-month gap between them. There was some turnover in physicians between the two study periods (~ 10%) that may have influenced the results; however, we did not observe any significant cultural, leadership, or general practice changes among the physicians between the two investigations that might have affected receptivity to A&F in general.

In our investigation, we were surprised to find that CT ordering increased during the study period for both the control and experimental groups. We are aware of no changes in the patient populations over the study period to explain this trend nor any other unmeasured influences to affect provider behavior in such a fashion. It is possible that our education regarding the potential adverse effects of CT imaging somehow had an inadvertent effect of increasing ordering behavior by influencing the clinicians’ decision-making around the risks and benefits of CT ordering. We believe this latter scenario to be unlikely given that the content of the education focused on national professional organizations’ emphasis on CT ordering reduction. However, our investigation was not designed to measure the effectiveness of the educational components, so it remains a possibility. There was active participation by physicians during the CT education session reflective of general engagement. However, individual engagement was not measured either for the in-person education session or for the associated slides sent out subsequently. So, while we believe it to be unlikely given the randomization methodology we employed, it remains possible that there was an unmeasured difference between the control and A&F arms related to the impact of the education session itself.

While our investigation included multiple diverse EDs, the physicians all worked for a single department, with shared central leadership. Therefore, there may be inherent limitations in the generalizability of our results. The directionality of the observed poor provider self-perception of ordering behavior in our study population compared to that of Kadhim-Saleh et al. [11] highlights this potential limitation. Nonetheless, it remains unclear if the self-perception pattern of our study cohort is representative of ED physicians broadly. Within our study population, it remains possible that the control and experimental arms exhibited different baseline perceptions as this was not measured for the control group. We believe this to be unlikely given that we employed randomization to minimize differences in baseline characteristics between the intervention and control arms. It is possible that the self-estimation itself somehow acted as an intervention, beyond the A&F. As the subjects were all part of a single department, there also was a possibility of cross-contamination between the control and intervention groups if subjects shared their experiences with each other. In practice, study participants, given their role as attending emergency physicians, would not have comanaged patients, except during hand-off of patient care from one to another at shift changes. As standard practice within the study group, sign-out (hand-off from one physician to another) of patients with active care in progress occurred only for a modest number of patients, and patients who may have been considered for a CT scan would have been an even smaller proportion of those patients (although this was not possible to measure specifically). Given the nature of sign-out practice, decisions to order CTs or not generally would not have occurred during the brief time of transfer of care of a patient; however, there was some potential for cross-contamination as on-coming physicians receiving patient sign-out may have gained some insight into the practice of their colleagues providing the patient sign-out. In addition, the trial itself may have been discussed informally among participants, for example, by email or in passing, and a cluster-randomized trial design would have avoided potential cross-contamination. Finally, the use of peer benchmarking and the absence of an ideal level of CT ordering (in contrast to clear “less is better” guidance in our prior opioid prescribing A&F intervention [16]) may have diminished the effectiveness of the A&F intervention in this case.

## Conclusions

Notwithstanding the potential limitations of our investigation’s methodology and the potential limitations in the comparison of our results to the prior study by Michael et al., the results of our study underline the importance of understanding physicians’ self-perception of performance before implementing an A&F intervention. Such an understanding can help to inform potential tailoring of how data are presented and, indeed, to decide whether A&F is the most appropriate intervention for a particular practice change. Future studies should explore the accuracy of self-perception of clinicians around a broader range of targeted behaviors (other types of test ordering and prescribing, screening, guideline adherence, referrals) and examine its potential moderating effect on the effectiveness of A&F interventions. Furthermore, it remains to be seen whether the accuracy of self-estimates might affect A&F’s effectiveness in other, non-emergency, and non-healthcare settings.

## Figures and Tables

**Fig. 3:**
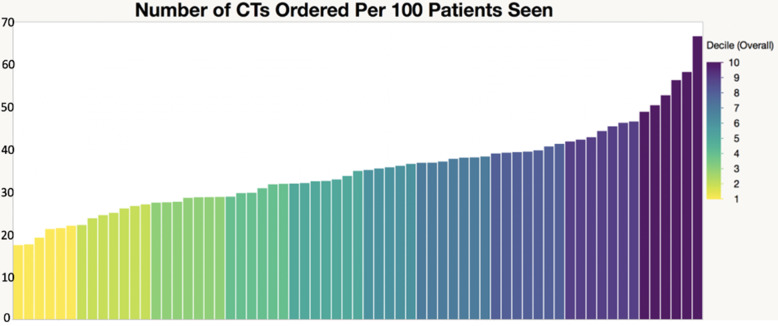
Graphical representation of CT ordering used in intervention

## Data Availability

The datasets analyzed during the current study are available from the corresponding author on reasonable request.
